# Isoliquiritigenin inhibits colorectal cancer progression by targeting the FGFR4/FASN mediated lipid metabolism pathway

**DOI:** 10.7150/jca.116357

**Published:** 2025-09-25

**Authors:** Xiaohui Zhai, Huizhi Yang, Zhiqin Tan, Song Wu, Qing Bao, Jian Liang, Hailin Tang

**Affiliations:** 1 Department of Medical Oncology, Guangdong Provincial Key Laboratory of Colorectal and Pelvic Floor Diseases, the Sixth Affiliated Hospital of Sun Yat-Sen University, Guangzhou 510655, China.; 2 Department of Gynaecology and Obstetrics, Air Force Hospital of the Southern Theater Command of the Chinese People's Liberation Army, Guangzhou, China.; 3 Department of Gynaecology and Obstetrics, Air Force Hospital of the Southern Theater Command of the Chinese People's Liberation Army, China.; 4 State Key Laboratory of Oncology in South China, Guangdong Provincial Clinical Research Center for Cancer, Sun Yat-Sen University Cancer Center, Guangzhou, China.; 5 Pharmaceutical Sciences, State Key Laboratory of Traditional Chinese Medicine Syndrome Guangzhou University of Chinese Medicine, Guangzhou, Guangdong 510006, China.

**Keywords:** colorectal cancer, isoliquiritigenin, FGFR4, FASN, PI3K/Akt

## Abstract

Colorectal cancer (CRC) is one of the most common malignant tumors. Isoliquiritigenin (ISL), a natural chalcone compound extracted from the roots of licorice and other plants, has demonstrated significant anti-tumor activity in various cancers. However, its specific mechanisms of action against CRC remain unclear. In this study, we investigated the molecular mechanisms underlying the effects of ISL targeting Fibroblast Growth Factor Receptor 4 (FGFR4) in CRC. Our findings revealed that FGFR4 is highly expressed in CRC cell lines, and functional assays demonstrated that silencing FGFR4 significantly inhibits cellular proliferation and migration. Further mechanistic studies showed that FGFR4 regulates fatty acid biosynthesis and the PI3K/Akt signaling pathway, as evidenced by the downregulation of Fatty Acid Synthase (FASN) and PI3K/Akt pathway proteins upon FGFR4 knockdown. Moreover, ISL significantly suppresses CRC cell proliferation and migration while disrupting tumor cell fatty acid metabolism. This study suggests that ISL may inhibit CRC progression by downregulating FGFR4 and suppressing PI3K/Akt-mediated fatty acid metabolism reprogramming.

## 1. Introduction

Colorectal cancer (CRC) is the second leading cause of cancer-related deaths worldwide, accounting for nearly 900,000 deaths annually^1^. While the incidence of CRC has stabilized or even declined in developed countries, it continues to rise in developing nations, making it the leading cause of cancer death in men, with a mortality rate of approximately 8.9% (28,700 of 322,800 cases), and the second leading cause in women, with a mortality rate of about 8.4% (24,310 of 288,920 cases)^1^. By 2035, the global incidence of CRC is projected to reach 2.5 million cases^2^, ^3^. The etiology of CRC is multifactorial, encompassing genetic, lifestyle, and environmental factors^4^, ^5^. Common risk factors include hereditary cancer syndromes, high-fat and low-fiber diets, chronic inflammatory bowel disease, obesity, smoking, and physical inactivity^6^, ^7^. Current treatment modalities for CRC have notable limitations: surgical resection often fails to eliminate micrometastases and is associated with a high rate of complications; chemotherapy and targeted therapies are frequently undermined by drug resistance and severe side effects^8^; radiotherapy is restricted to specific patient populations and carries significant toxicity; and immunotherapy is effective for only a small subset of patients, with highly variable responses^9^, ^10^. Although combination therapies can enhance efficacy, they are often hindered by cumulative toxicity and the complexity of treatment coordination, limiting patient tolerance and outcomes^11^. These challenges underscore the need for optimized therapeutic strategies^12^, ^13^. The development of novel formulations with enhanced release control and targeted delivery may improve therapeutic efficacy while minimizing the toxicity associated with conventional chemotherapy drugs. As a result, the exploration of natural products for the prevention and treatment of colorectal cancer has emerged as a key focus in cancer research^14^-^16^.

Studies have suggested that estrogen may play a potential role in the prevention and treatment of CRC^17^. Phytoestrogens, a class of non-steroidal plant-derived compounds, can mimic the effects of estrogen in the body^18^. Isoliquiritigenin, a flavonoid extracted from the rhizomes of the traditional Chinese medicinal herb licorice, exhibits various biological activities, including antioxidant, anti-inflammatory, and anti-tumor properties^19^, ^20^. Research has shown that isoliquiritigenin protects the cardiovascular system in cardiovascular diseases through its antioxidant and anti-inflammatory effects^21^, enhances insulin sensitivity in diabetes^22^, suppresses inflammatory cytokines in chronic inflammatory disorders^23^, and alleviates oxidative stress and neuroinflammation in neurodegenerative diseases such as Alzheimer's^24^. In the field of cancer research—including CRC^25^, breast cancer^26^, ^27^, glioma^28^, and lung cancer^29^—it demonstrates anti-tumor effects by inhibiting cell proliferation, invasion, and angiogenesis, as well as promoting apoptosis. Its anti-tumor activity against CRC is increasingly being recognized, however, the precise mechanisms underlying its effects on CRC remain unclear.

Metabolic reprogramming is a hallmark of cancer, enabling tumor cells to adjust their metabolic networks to meet the demands for rapid proliferation, growth, and invasion. These metabolic alterations not only help tumor cells adapt to their microenvironment but also actively promote cancer progression^30^. Lipid metabolism reprogramming is a critical component of these changes, involving processes such as fatty acid synthesis, breakdown, storage, and transport^31^, ^32^. Due to the pressures of rapid proliferation and survival, tumor cells exhibit an increased demand for fatty acids. To meet this requirement, they often upregulate the expression of key enzymes such as fatty acid synthase (FASN)^33^ and acetyl-CoA carboxylase (ACC)^34^, thereby enhancing endogenous fatty acid synthesis.

This study aims to evaluate the involvement of isoliquiritigenin (ISL) in lipid metabolism and its impact on colorectal cancer (CRC) cell proliferation and invasion. The results demonstrate that ISL inhibits the proliferation and invasion of CRC cells in vitro, with the potential mechanism involving downregulation of the PI3K/AKT pathway and suppression of FGFT4-dependent lipid metabolism.

## 2. Methods and Materials

### 2.1 Cell culture and treatment

Colorectal cell lines NCM460, SW480, SW620, HCT-116, HT-29, and LOVO were purchased from ATCC and cultured in media containing 10% fetal bovine serum (FBS), penicillin (100 U/mL), and streptomycin (100 μg/mL) at 37°C and 5% CO₂. FGFR4 expression in SW480 and SW620 cells was stably knocked down using FGFR4-targeting shRNA from Umine Biotechnology Co., Ltd. (Guangzhou, China), with knockdown efficiency confirmed by RT-PCR and Western blot.Isoliquiritigenin (ISL) was obtained from Sigma-Aldrich, dissolved in DMSO at 100 mM, and diluted to working concentrations with culture medium before use. Control cells received equivalent volumes of DMSO.

### 2.2 Cell Counting Kit-8 assay

Cell proliferation was assessed using the CCK-8 assay (UElandy, China) following the manufacturer's protocol. Briefly, cells were seeded into 96-well plates at a density of 2000-3000 cells/well and treated with different conditions for the indicated times. At each time point, 10 μL of CCK-8 reagent was added to each well and incubated for 1-2 hours at 37°C. Absorbance at 450 nm was measured using a microplate reader. Each experiment was performed in triplicate.

### 2.3 Cell invasive assay

The invasive capacity of cells was evaluated using 24-well Transwell chambers (Corning, USA) with Matrigel coating. Cells (5 × 10⁴) suspended in serum-free medium were added to the upper chamber, and the lower chamber was filled with medium containing 10% FBS. After 24 hours of incubation at 37°C, non-invading cells were removed, and invading cells on the lower membrane surface were fixed in 4% paraformaldehyde, stained with crystal violet, and counted under a microscope in five randomly selected fields.

### 2.4 Quantitative real-time PCR

Total RNA was extracted using TRIzol reagent (Invitrogen, USA) and reverse-transcribed into cDNA using a reverse transcription kit (Takara, Japan). qRT-PCR was performed using SYBR Green Master Mix (Roche, Switzerland) on a Bio-Rad detection system. Relative mRNA expression levels were calculated using the 2⁻ΔΔCt method^35^, with GAPDH as an internal control. Primers used for PCR are listed in Supplementary Table S1.

### 2.5 Western blotting

Cells were lysed in RIPA buffer containing protease and phosphatase inhibitors (Beyotime, China), and protein concentration was determined using a BCA assay kit (Thermo Fisher, USA). Equal amounts of protein (20-30 μg) were separated by SDS-PAGE and transferred onto PVDF membranes (Millipore, USA). Membranes were blocked in 5% non-fat milk for 1 hour at room temperature and incubated with primary antibodies overnight at 4°C^36^, ^37^. The primary antibodies used include GAPDH (#2118, 1:1000), FGFR4 (#8562, 1:1000), FASN (#3180, 1:1000), Phospho-Akt (Ser473) (#4060, 1:2000), PI3 Kinase p85 (#4292, 1:1000), PI3 Kinase (#4249, 1:1000), and Akt (#9272, 1:1000) (all purchased from CST). After washing, membranes were incubated with mouse or rabbit secondary antibodies (purchased from Proteintech) for 1 hour at room temperature. Protein bands were visualized using enhanced chemiluminescence (ECL) reagent (Bio-Rad, USA) and imaged with a ChemiDoc system. GAPDH was used as a loading control.

### 2.6 Oil red O staining

Lipid droplet accumulation in tumor cells was analyzed using Oil Red O staining. Briefly, cells cultured in 12-well plates were fixed with 4% paraformaldehyde, stained with Oil Red O solution, and observed under a microscope. The stain was extracted for quantitative analysis at 520 nm using a spectrophotometer. Confocal microscopy was used for imaging lipid droplet distribution.

### 2.7 Fatty acid oxidation assay

To evaluate the fatty acid oxidation (FAO) rate, tumor cells were seeded into 6-well plates and cultured until reaching approximately 80% confluency. Cells were incubated in a medium supplemented with ^14^C-labeled fatty acids. All procedures were performed following the manufacturer's instructions for the assay kit (BR00001).

### 2.8 Measurement of total fatty acid content

The total fatty acid content in tumor cells was determined using Free Fatty Acid Colorimetric Assay Kit (ab282927). Fatty acids were derivatized into their methyl esters (FAMEs) and quantified against internal standards.

### 2.9 RNA-seq analysis

We performed transcriptome sequencing using SW480 colorectal cancer cell lines with FGFR4 knockdown. Total RNA was extracted from the cells using TRIzol reagent, then were sent to Ruibo(Guangzhou, China). Differentially expressed genes (DEGs) were identified based on criteria of |Log2 fold change| ≥ 2 and an adjusted *p*-value < 0.05. Further enrichment analyses were conducted to explore the involved biological pathways and mechanisms.

### 2.10 Statistical analysis

All experiments were performed at least three times, and the resulting data were analyzed using GraphPad Prism 9 software. Results are presented as mean ± standard deviation (SD). Comparisons between two groups were conducted using Student's t-test, with statistical significance defined as *p<* 0.05.

## 3. Results

### 3.1 Knockdown of FGFR4 suppresses colorectal cancer cell proliferation and invasion

**Figure 1:**
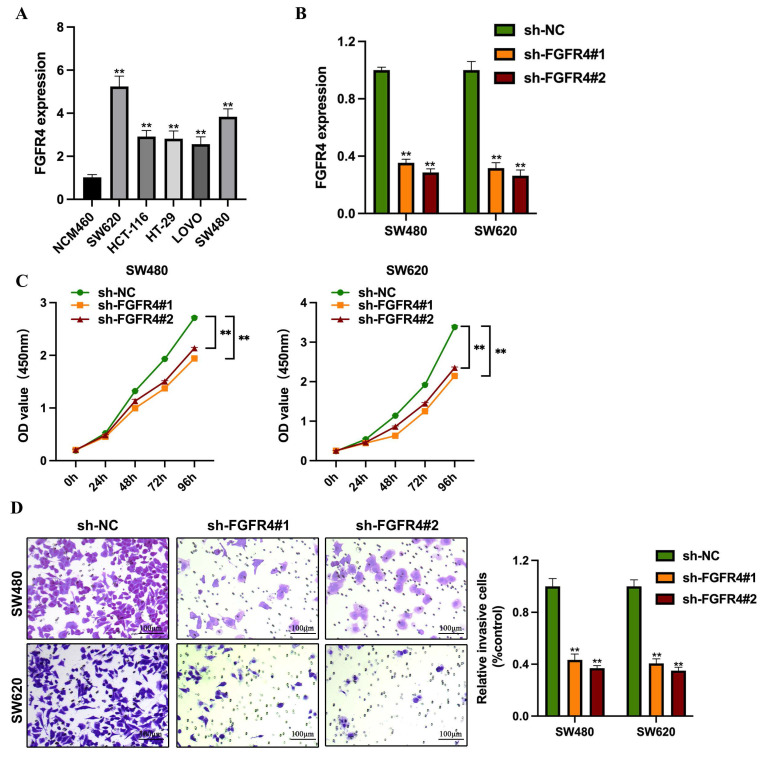
Knockdown of FGFR4 suppresses colorectal cancer cell proliferation and invasion. (A) RT-qPCR analysis revealed FGFR4 expression levels across various colorectal cancer cell lines. (B) Stable shRNA knockdown cell lines (sh-FGFR4#1 and sh-FGFR4#2) were generated in SW480 and SW620 colorectal cancer cell lines, and FGFR4 expression levels were confirmed. (C) CCK-8 assays demonstrated significant growth inhibition in SW480 and SW620 cells over 1-6 days following FGFR4 knockdown. (D) Transwell assays showed reduced invasion capacity of SW480 and SW620 cells after FGFR4 knockdown. **P <0.05.

FGFR4 expression was assessed across colorectal cancer cell lines representing various molecular subtypes. RT-qPCR analysis revealed significantly higher levels of FGFR4 in colorectal cancer cells compared to normal intestinal epithelial cells (NCM460) (Figure 1A). Among these, SW480 and SW620 exhibited the highest FGFR4 expression levels, prompting their selection for FGFR4 knockdown experiments (Figure 1B). In CCK-8 assays, the proliferation capacity of the FGFR4-knockdown group was significantly reduced compared to the control group (p < 0.05) (Figure 1C). Furthermore, Transwell assays demonstrated that the invasive ability of FGFR4-knockdown cells was also markedly lower than that of the control group, indicating that FGFR4 knockdown inhibits the invasiveness of colorectal cancer cells (Figure 1D).

### 3.2 Knockdown of FGFR4 inhibits fatty acid metabolism in colorectal cancer

**Figure 2:**
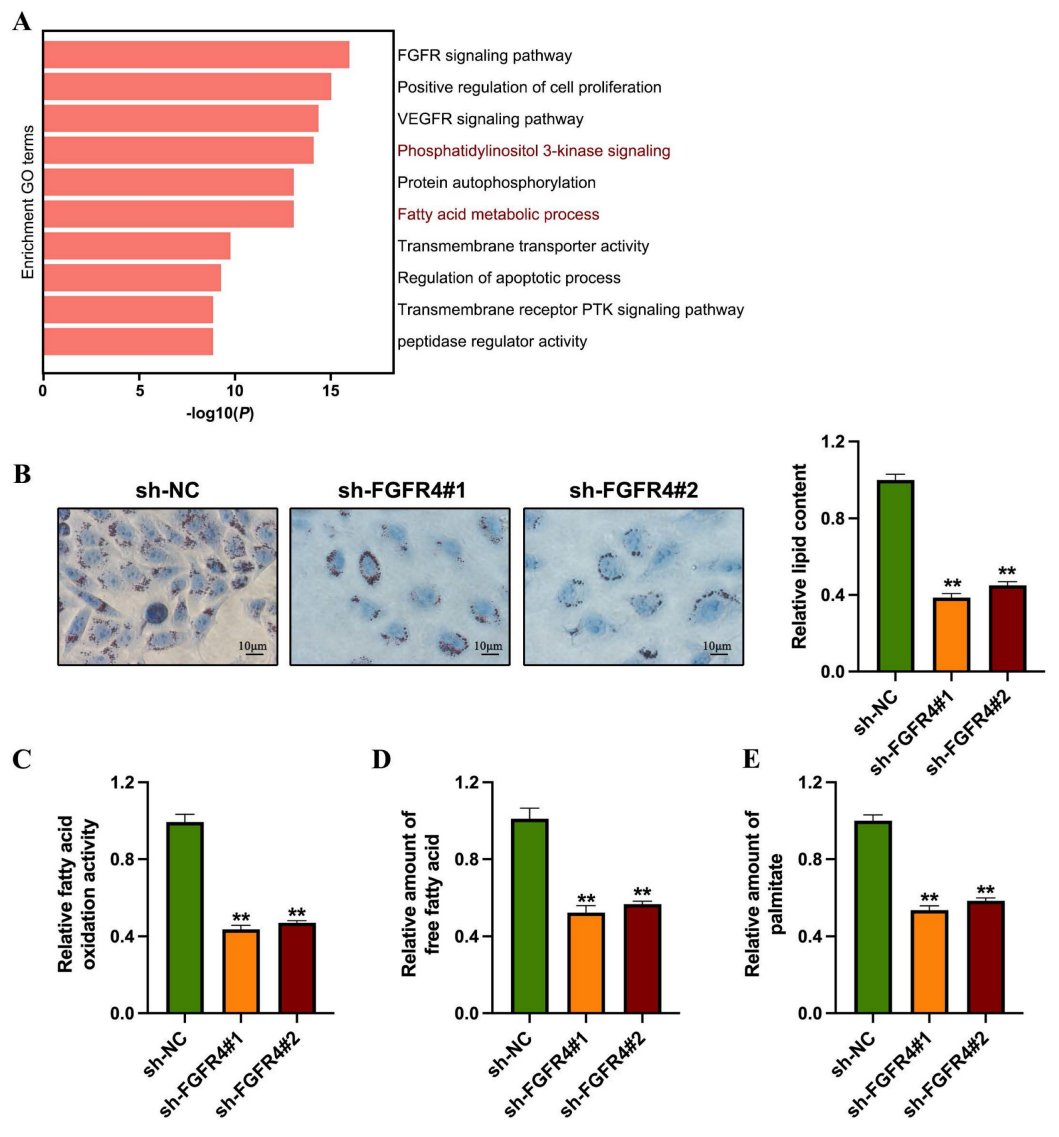
Knockdown of FGFR4 inhibits fatty acid metabolism in colorectal cancer. (A) Network analysis using Metascape highlighted fatty acid biosynthesis and the PI3K-Akt signaling pathway as critical processes regulated by FGFR4. (B) Oil Red O staining showed decreased lipid droplet synthesis in sh-FGFR4 cells. (C) Quantification of total free fatty acid content in SW480/sh-NC and SW480/sh-FGFR4 cells. (D) Measurement of fatty acid oxidation activity in SW480/sh-NC and SW480/sh-FGFR4 cells. (E) Quantification of palmitic acid levels in SW480/sh-NC and SW480/sh-FGFR4 cells. ** p< 0.05.

To investigate the role of FGFR4 in the progression of colorectal cancer, we conducted transcriptomic sequencing analysis using the FGFR4-stably silenced SW480 colorectal cancer cell line. Differential expression analysis identified 867 upregulated genes and 793 downregulated genes between groups. Network pathway clustering further revealed that fatty acid biosynthesis and the PI3K-Akt signaling pathway are central among the pathways regulated by FGFR4, suggesting its involvement in both fatty acid metabolism and PI3K-Akt signaling (Figure 2A). Subsequent lipid metabolism experiments showed that, compared to control cells, sh-FGFR4 cells exhibited reduced lipid droplet accumulation (Figure 2B), decreased fatty acid oxidation rate (Figure 2C), and lower overall fatty acid content (Figure 2D). Additionally, quantitative analysis of cellular palmitic acid levels demonstrated that FGFR4 knockdown significantly reduced palmitic acid levels in SW480 cells (Figure 2E).

### 3.3 FGFR4 promotes fatty acid metabolism in colorectal cancer by upregulating FASN

**Figure 4:**
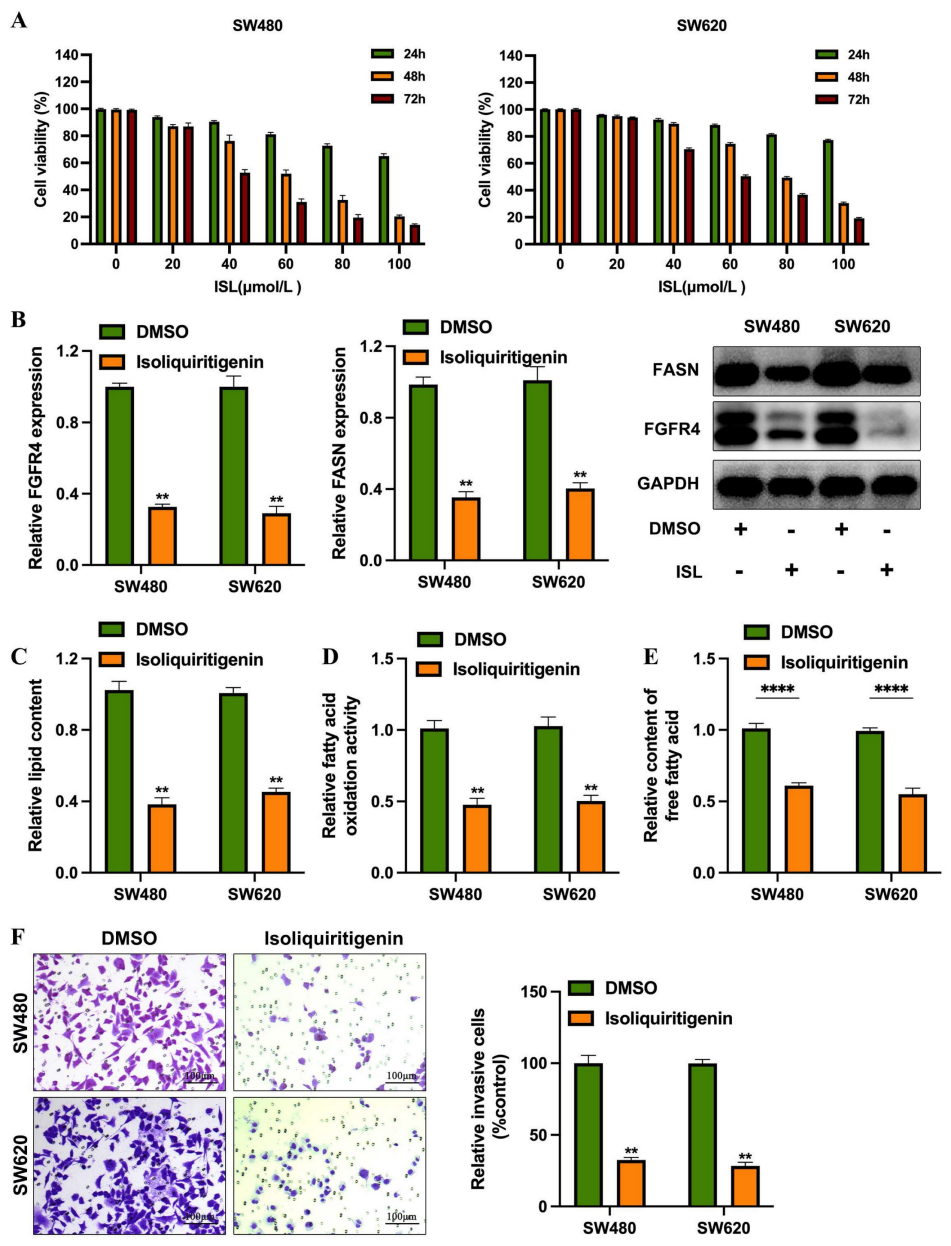
ISL Effectively inhibits colorectal cancer cell invasion by suppressing fatty acid metabolism. (A) The CCK-8 assay revealed that ISL treatment for 24, 48, and 72 hours significantly inhibited the viability of SW480 and SW620 cells. (B) Western blot analysis demonstrated that ISL treatment for 24 and 48 hours reduced the expression levels of FGFR4 and FASN proteins in colorectal cancer cells. (C) Oil Red O staining indicated decreased lipid droplet formation in colorectal cancer cells following ISL treatment. (D) Quantitative analysis showed a reduction in total free fatty acid content in ISL-treated colorectal cancer cells compared to control cells. (E) ISL treatment also led to a decrease in fatty acid oxidation activity in colorectal cancer cells, as evaluated by quantitative assays. (F) Transwell assays confirmed that ISL treatment inhibited the invasive capabilities of SW480 cells. ** *p<* 0.05.

To further investigate the downstream effector proteins of FGFR4, genes downregulated in FGFR4-knockdown cells were intersected with differentially expressed genes identified from TCGA-COAD datasets comparing high FGFR4 expression vs. low FGFR4 expression, as well as with genes associated with fatty acid biosynthesis. Fatty acid synthase (FASN) emerged as the sole overlapping gene (Figure 4A). Analysis of the TCGA-COAD database revealed a positive correlation between FGFR4 and FASN expression levels (Figure 4B). qRT-PCR and Western blot analysis demonstrated that FGFR4 knockdown in SW480 and SW620 cells resulted in a concurrent reduction in FASN expression (Figures 4C-D). Furthermore, treatment of SW480 cells with the FGFR4-specific inhibitor Roblitinib reduced PI3K and Akt phosphorylation, as well as FABP5 expression (Figure 4E). These findings suggest that FGFR4 facilitates fatty acid metabolism remodeling by activating the downstream PI3K-Akt pathway and upregulating FASN expression, thereby promoting the initiation and progression of colorectal cancer.

### 3.4 ISL effectively inhibits colorectal cancer cell invasion by suppressing fatty acid metabolism

To evaluate the clinical potential of isoliquiritigenin (ISL) in colorectal cancer (CRC), researchers conducted in vitro experiments using the SW480 and SW620 cell lines treated with ISL at concentrations ranging from 0-100 μmol/L for 24-72 hours. Results showed that ISL significantly inhibited cell proliferation in a dose- and time-dependent manner. In SW480 cells, the half-maximal inhibitory concentrations (IC50) at 24, 48, and 72 hours were 148.2 μmol/L, 60.37 μmol/L, and 42.95 μmol/L, respectively. Similarly, SW620 cells showed IC50 values of 240.8 μmol/L, 79.56 μmol/L, and 59.81 μmol/L at the same time points (Figure 4A). Based on the IC50 values at 48 hours (60.37 μmol/L for SW480 and 79.56 μmol/L for SW620), subsequent studies were conducted using ISL concentrations of 50 μmol/L for SW480 cells and 70 μmol/L for SW620 cells.

Treatment with ISL resulted in a significant reduction in the expression levels of FGFR4 and FASN in both SW480 and SW620 cells (Figure 4B). Further experiments revealed that ISL decreased lipid droplet formation (Figure 4C), total fatty acid content (Figure 4D), and fatty acid oxidation levels (Figure 4E), indicating its role in reprogramming lipid metabolism in CRC cells. Additionally, functional assays demonstrated that ISL suppressed the invasive capabilities of SW480 and SW620 cells (Figure 4F). hese findings suggest that isoliquiritigenin effectively inhibits CRC progression by targeting FGFR4 and disrupting lipid metabolism.

## 4. Discussion

Fibroblast Growth Factor Receptor 4 (FGFR4) has garnered attention as a key oncogenic driver in multiple malignancies, including liver, breast, lung cancers and CRC^38^-^40^. Recent studies have implicated FGFR4 in regulating proliferation, migration, and resistance to therapies through its downstream signaling cascade^41^. In CRC, our study confirms that FGFR4 is highly upregulated in tumor cells, consistent with prior data correlating FGFR4 expression with poor prognosis^40^. FGFR4's ability to activate PI3K/Akt signaling is particularly noteworthy, as this pathway orchestrates cellular processes such as growth and survival, while also playing a central role in lipid metabolism. These findings align with previous research emphasizing PI3K/Akt as a master regulator of metabolic reprogramming, wherein tumor cells shift towards heightened fatty acid synthesis to sustain rapid growth and invasion. Similarly, Guo et.al identified KDM6A as a driver of HCC progression via FGFR4-mediated activation of the PI3K-AKT-mTOR pathway, leading to altered glucose and lipid metabolism^42^. Besides, FGFR4 drives basal-like breast cancer cell survival through PI3K/AKT activation, with a subset relying on FGF19-mediated autocrine signaling^43^.

Fatty acid metabolism is increasingly recognized as a hallmark of cancer progression^44^. Tumor cells rely on enhanced lipogenesis, driven by enzymes such as FASN, to support membrane synthesis, energy production, and oncogenic signaling^45^, ^46^. Our data reveal FGFR4 as a key regulator of FASN expression, further implicating lipid biosynthetic pathways in CRC malignancy. Notably, FASN has also been identified as a therapeutic target in diverse cancer types, with its inhibition leading to tumor suppression^47^. Thus, the FGFR4-FASN axis presents a dual opportunity for both mechanistic insight and therapeutic intervention in CRC.

The role of ISL in modulating lipid metabolism provides a novel perspective in anti-cancer therapy. Current anti-metabolic strategies, such as inhibitors targeting FASN, ACC, or lipid transport proteins, face challenges including toxicity, off-target effects, and compensatory metabolic shifts^48^, ^49^. ISL, on the other hand, offers a natural and low-toxicity alternative, demonstrating the ability to downregulate FGFR4, suppress FASN, and inhibit the PI3K/Akt pathway—all key drivers of lipid metabolism and CRC progression. Combining ISL with existing treatments targeting FGFR or metabolic enzymes may further enhance therapeutic efficacy. Prior studies suggest synergies between FGFR or PI3K inhibitors and chemotherapeutic agents, especially in overcoming drug resistance^50^, ^51^. Despite significant progress, challenges remain. The translational application of ISL requires validation in in vivo models and clinical trials to assess its bioavailability, pharmacodynamics, and long-term effects. Furthermore, heterogeneity among CRC subtypes underscores the need for personalized approaches, especially in tumors reliant on distinct metabolic pathways. In addition, this study has not yet clarified the specific molecular mechanisms by which ISL regulates FGFR and FASN, which warrants further investigation in future research. Moreover, the current study is limited to in vitro experiments and lacks verification through in vivo animal models, highlighting the need for further validation in animal studies.

**Figure 5:**
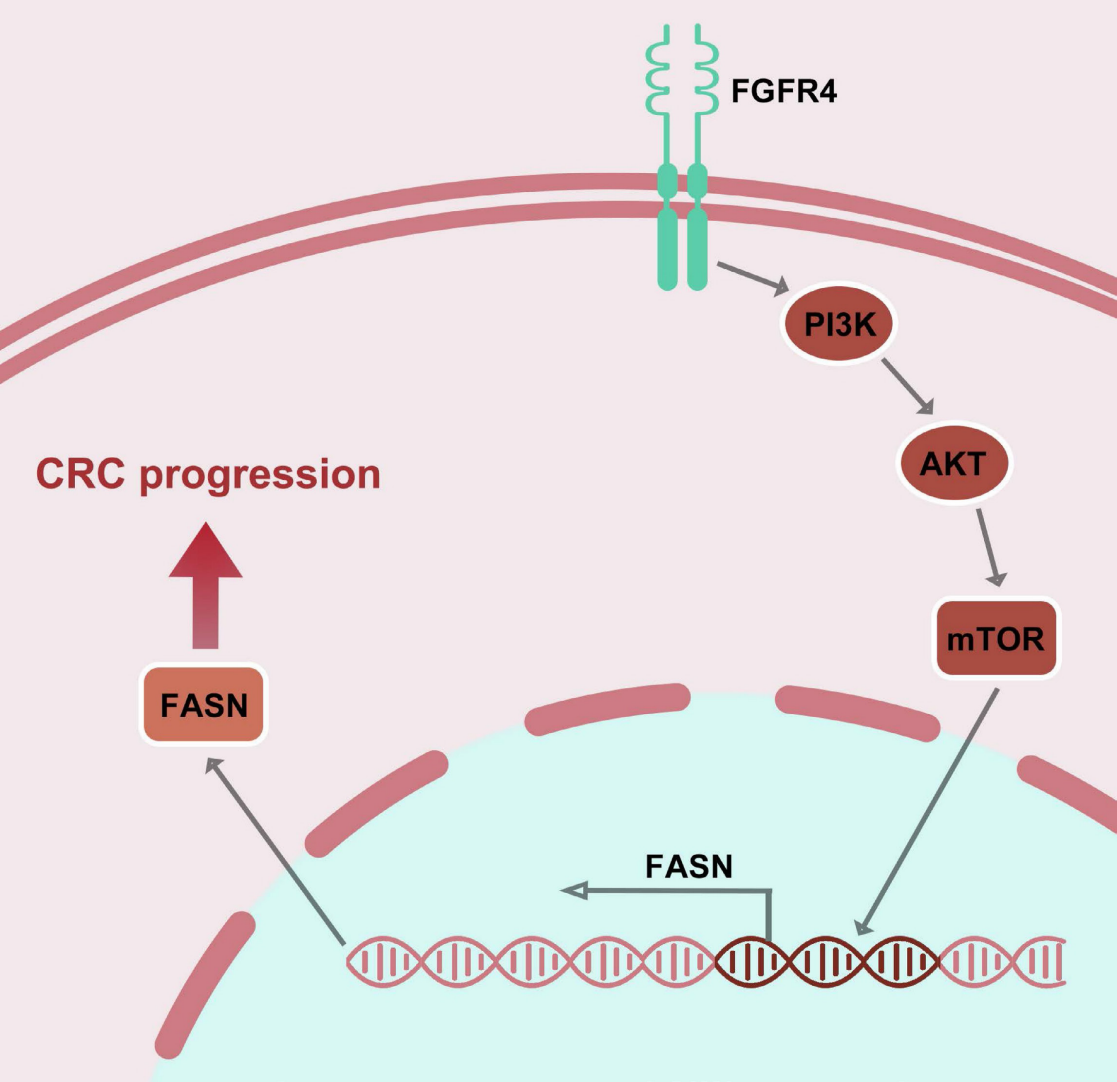
Mechanism of isoliquiritigenin inhibits colorectal cancer progression by targeting the FGFR4/FASN pathway.

In conclusion, this study identifies FGFR4 as a critical driver of lipid metabolic reprogramming in CRC and demonstrates that ISL effectively inhibits this axis, suppressing tumor progression (Figure 5). Future research integrating ISL with metabolic therapies and immunomodulation may pave the way for innovative treatments that improve outcomes for CRC patients.

## 5. Conclusion

This study reveals FGFR4 as a key driver of lipid metabolism in CRC and demonstrates that isoliquiritigenin (ISL) effectively inhibits tumor progression by targeting the FGFR4/FASN pathway. ISL shows promise as a novel metabolic therapy, warranting further exploration for clinical application.

## Supplementary Material

Supplementary tables.

## Figures and Tables

**Figure 3:**
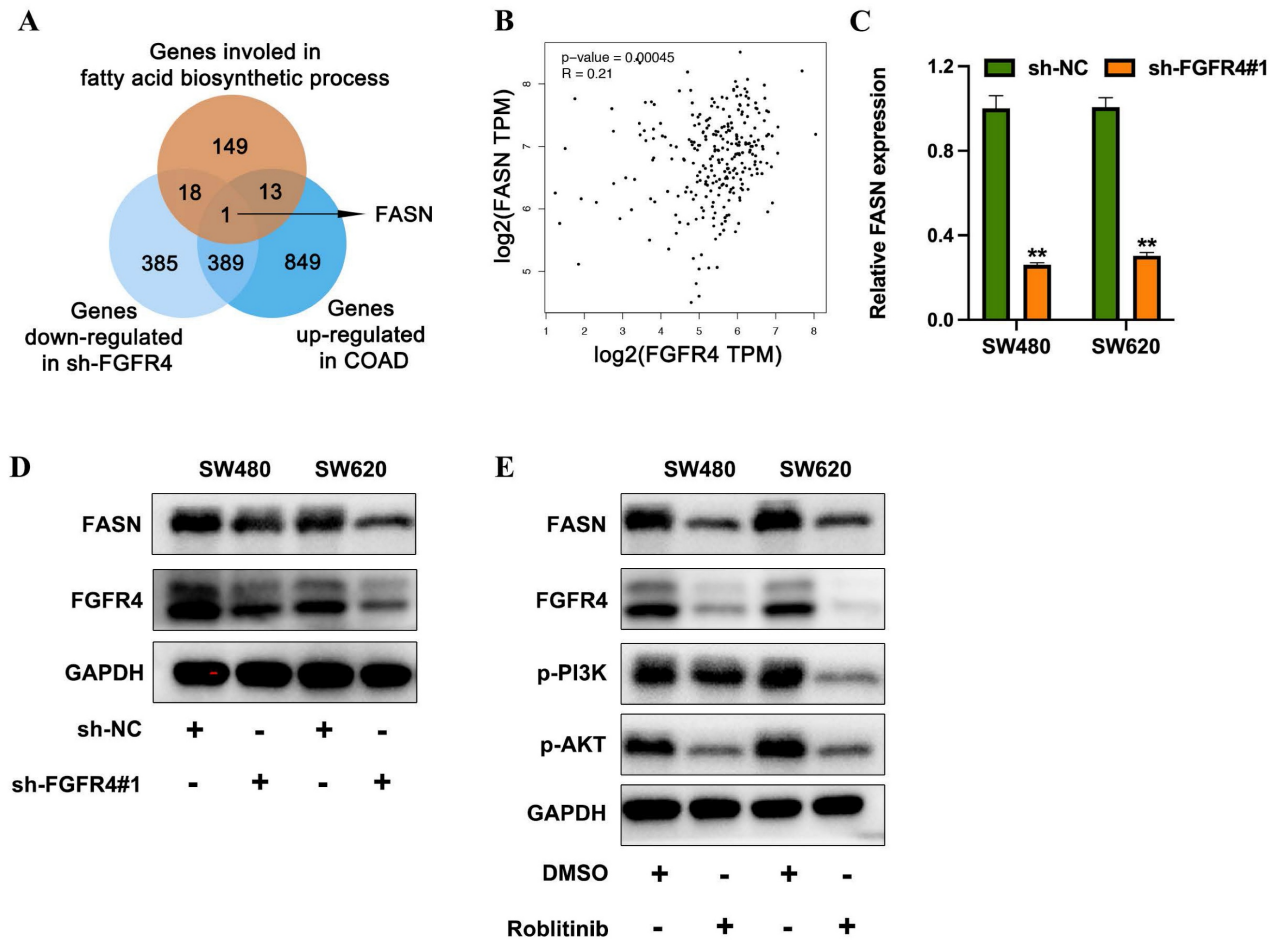
FGFR4 promotes fatty acid metabolism in colorectal cancer by upregulating FASN. (A) Venn diagram illustrating the overlap between genes downregulated in the sh-FGFR4 group, genes upregulated in the TCGA-COAD dataset, and genes involved in fatty acid biosynthesis. (B) Correlation analysis from the TCGA-COAD database showing the association between FGFR4 and FASN expression levels. (C) qPCR results demonstrating reduced FASN expression in SW480 and SW620 cells following FGFR4 knockdown. (D) Western blot analysis confirming decreased FASN protein levels in SW480 and SW620 cells after FGFR4 interference. (E) Protein level changes in phosphorylated and total PI3K, Akt, and FABP5 detected in colorectal cancer cells treated with FGFR4 inhibitor Roblitinib (5 µM). ** *p<* 0.05.
