# Supplementary material for: Isoliquiritigenin inhibits colorectal cancer progression by targeting the FGFR4/FASN mediated lipid metabolism pathway
Source: J Cancer. 2025 Sep 25;16(14):4071–80. doi: 10.7150/jca.116357 (PMC12595245; doi:10.7150/jca.116357)
Supplement: Supplementary file 1 — Supplementary tables. [file jcav16p4071s1.pdf]

Supplementary Table S1

FGFR4	Forward Primer	GCACTGCAGTCTCGTGATGG
	Reverse Primer	CCACAGCGTTCTCTACCAGG
FASN	Forward Primer	AACCGGCTCTCCTTCTTCTT
	Reverse Primer	TTGGGCTTCAGCAGGACATT
GAPDH	Forward Primer	CAACCGGGAAGGAAATGAAT
	Reverse Primer	CATCACCCCGAGGAGAAATC

| qRT-PCR Primers used in this study
